# Effectiveness of optimized food-based recommendation promotion to improve nutritional status and lipid profiles among Minangkabau women with dyslipidemia: A cluster-randomized trial

**DOI:** 10.1186/s12889-021-12462-5

**Published:** 2022-01-06

**Authors:** Gusnedi Gusnedi, Umi Fahmida, Fiastuti Witjaksono, Fariz Nurwidya, Muchtaruddin Mansyur, Ratna Djuwita, Cesilia Meti Dwiriani, Murdani Abdullah

**Affiliations:** 1 Department of Nutrition, Health Ministry Polytechnic of Padang, Padang, West Sumatra Indonesia; 2 Department of Nutrition, Faculty of Medicine, Universitas Indonesia – Dr. Cipto Mangunkusumo General Hospital, Jakarta, Indonesia; 3 Southeast Asian Ministers of Education Organization Regional Centre for Food and Nutrition (SEAMEO-RECFON) – Pusat Kajian Gizi Regional (PKGR) Universitas Indonesia, Jakarta, Indonesia; 4 Department of Community Medicine, Faculty of Medicine, Universitas Indonesia, Jakarta, Indonesia; 5 Department of Epidemiology, Faculty of Public Health, Universitas Indonesia, Jakarta, Indonesia; 6 Department of Community Nutrition, Faculty of Human Ecology, IPB University, Jakarta, Indonesia; 7 Division of Gastro-enterology, Department of Internal Medicine, Faculty of Medicine, Universitas Indonesia – Dr. Cipto Mangunkusumo General Hospital, Jakarta, Indonesia

**Keywords:** Linear programming, Food-based recommendation, Nutritional status, Dyslipidemia, Minangkabau women

## Abstract

**Background:**

In women of Minangkabau ethnicity, a high prevalence of dyslipidemia, overweight, and obesity is thought to be closely related to poor dietary practices. Promotion of local specific food-based recommendations (FBRs) was previously found to be effective in improving dietary practice and nutrient intakes related to dyslipidemia. This study aimed to describe the effects of the FBR promotion on the nutritional status and lipid profiles of Minangkabau women with dyslipidemia.

**Methods:**

We used a cluster-randomized design with a total subject of 123 Minangkabau women of reproductive age with dyslipidemia. They were recruited from 16 sub-villages and assigned to either the FBR group (n = 61) or the non-FBR group (n = 62). Data on body weight, height, waist circumference, and lipid profiles were collected at the baseline and the end of the trial. Linear mixed model analysis was used to analyze the effect of the intervention on nutritional status and lipid profiles.

**Results:**

The mean effect (95% confidence interval) of the intervention on body weight, body mass index, and waist circumference for the FBR group versus the non-FBR group were -1.1 (-1.8; -0.39) kg, -0.43(-0.76; -0.11) kg/m2 and -2.1(-3.7;-0.46) mm respectively (p <0.05). The Castelli’s index in the FBR group improved, but there was no significant between-group difference in the change of total cholesterol, LDL cholesterol, HDL cholesterol, and triglycerides at the end of the intervention.

**Conclusion:**

The promotion of the FBRs positively impact the nutritional status but did not significantly affect the blood lipid profile of Minangkabau women with dyslipidemia**.**

**Trial Registration:**

The trial was retrospectively registered at ClinicalTrials.gov Protocol Registration and Result System (PRS) as NCT04085874, in September 2019.

## Background

Dyslipidemia and obesity are among the major risk factors for certain non-communicable diseases, such as heart disease and stroke [1]. The results of the latest National Health Survey showed that the prevalence of dyslipidemia in Indonesia, based on abnormal total cholesterol (TC) and low-density lipoprotein (LDL) levels, was approximately 30% and 74%, respectively. A higher prevalence of dyslipidemia was found among women as compared with men. Additionally, the prevalence of overweight and obesity among women has reached 53% (defined as a body mass index [BMI] of ≥25.0), whereas the prevalence of abdominal obesity is approximately 46% (defined as a waist circumference of ≥80 cm) [2]. An earlier study found that, in Indonesia, Minangkabau women had higher mean TC levels than individuals of other ethnicities [3]. Our latest study among Minangkabau women of reproductive age also found that the prevalence of dyslipidemia based on LDL level was relatively high (approximately 44%), and most of these women (approximately 70%) were overweight or obese [4].

The high prevalence of dyslipidemia, overweight, and obesity among women of Minangkabau ethnicity is believed to be closely related to poor dietary practices [3]. Approximately 20.7% of energy intake comes from saturated fat (>10%), with a polyunsaturated fatty acid to saturated fat ratio of only 0.15 [5]. The use of other saturated fat sources, palm oil, and animal resource protein among the Minangkabau population is considerably high because of their traditional heritage of food processing [6]. On average, a household with three or four children uses 250 g of cooking oil each day [7]. According to data from the Indonesian Total Diet Survey, the Minangkabau population consumes on average 50.4 g of fat daily in the form of cooking oil, coconut oil, and coconut and <100 g of vegetables and fruits per day [8].

To promote healthy eating for chronic disease prevention, a linear programming (LP) approach can be used effectively to translate nutritional requirements into local-specific food-based recommendations (FBRs) [9, 10]. .Using LP, the World Health Organization has developed user-friendly diet modeling software called Optifood [11]. In our recent study, we developed locally specific FBRs by translating national dietary guidelines into the local context, based on the habitual dietary patterns and nutritional requirements of Minangkabau women with dyslipidemia [4]. However, further efforts are required to ensure the adoption of the FBRs by the target community to ultimately achieve positive impacts on health. Therefore, in our community-based trial, we investigated the effect of FBR promotion on dietary practices, nutrient intake, nutritional status, and lipid profiles. The trial convincingly showed positive impacts on dietary practices and nutrient intakes [12]. Meanwhile, this article is intended to describe the effects of FBR promotion on nutritional status and lipid profiles. We hypothesized that individuals in the intervention group would demonstrate better improvement in nutritional status and lipid profile than those in the comparison group.

## Methods

### Study design and subjects

This study is part of a clustered-randomized community-based trial under the title “Effect of Food-Based Recommendation Promotion on Dietary Practices, Nutrient Intakes, Nutritional Status, and Lipid Profiles among Minangkabau Women with Dyslipidemia.” It was conducted in four selected health centers in Padang City, the capital of West Sumatra, Indonesia. Potential subjects were identified before dyslipidemia screening. The inclusion criteria for subject recruitment were as follows: women of reproductive age (20–44 years), native Minangkabau ethnicity (i.e., both father and mother of Minangkabau ethnicity), abnormal blood lipid profile (TC > 200 mg/dL, LDL cholesterol > 100 mg/dL, high-density lipoprotein [HDL] cholesterol < 60 mg/dL, or triglycerides [TG] > 150 mg/dL), and ability to sign written informed consent. The exclusion criteria include pregnancy; current or history of smoking or alcoholism; history of heart disease, diabetes, asthma, cancer, chronic digestive tract disorders, hemophilia, or other chronic diseases; routinely taking cholesterol-lowering or blood pressure medications; being vegetarian; using estrogen therapy; and participating in other studies. When entering the treatment phase, dropout criteria were applied if (1) the research subjects did not participate in an education session for three consecutive weeks, (2) an indication of exclusion criteria was found in the subject during the study (e.g., being pregnant, newly diagnosed with a chronic disease), (3) research subjects did not undergo a complete blood examination, or (4) a subject desired to withdraw from the intervention.

We recruited 123 subjects living in 16 sub-villages of the four selected health centers. Based on a previous study [13], the sample size was calculated to detect the expected mean difference in LDL cholesterol level of 14 mg/dL, with 20 mg/dL standard deviation, at 80% power and a 5% level of significance. The final sample size of 60 subjects for each group intervention accommodated a 20% dropout rate and adjustment for the cluster effect (multiplied by 1.5). The subjects were assigned into two groups in a 1:1 allocation ratio. The intervention group (FBR group, n = 61) received 12 weeks of FBR promotion, which emphasized ways to eat better based on the local food-based balanced nutrition guidelines, whereas the comparison group (non-FBR group, n = 62) received regular nutritional counseling based on usual practice, without specific emphasis on the messages in the FBR.

### Randomization

To avoid contamination and to eliminate access barriers to participation in FBR promotion, randomization was conducted before subject recruitment at the health center level. The four health centers in the subdistricts of Koto Tangah and Nanggalo were randomly assigned to the intervention or comparison group using opaque envelopes by a public officer who was not involved in the present study. The environmental factors of all four health centers are similar, including food availability, access to transportation, public services, and facilities. Blinding was not possible because both the subjects and the researchers clearly understood the differences between the two groups.

### Intervention

The optimized FBR development process was described in our previous study [4]. The intervention used the theory of self-monitoring approach in nutrition promotion [13]. The participants in the FBR group were encouraged to become aware of aspects of dyslipidemia regarding their current diet. They were then introduced to the FBRs and asked to practice them in their daily diet. Briefly, as previously mentioned [4, 12], the FBR comprised the following messages:

1. Consume two to three main meals with one to two servings of healthy snacks every day.
2. Consume two to three servings of protein every day, including a minimum of five servings of fish per week, two to three servings of eggs per week, two to three servings of poultry per week, and at least seven servings of soy products per week.
3. Consume at least two servings per day of vegetables, including five servings per week of dark green leafy vegetables such as cassava leaf, spinach, and kale.
4. Consume at least one serving per day of fruit such as guava, banana, papaya, watermelon, and sweet orange.
5. Consume at least five servings per week of potatoes (e.g., small potatoes in chicken rendang and mashed potatoes with eggs)
6. Limit fried foods or foods cooked with coconut milk to a maximum of two servings per day.

The promoters taught the participants to plan their diet changes and set targets to achieve. In line with messages in the FBR, the main goals set for the intervention were: 1) to control frequency consumption and portion size of staple foods and snacks; 2) to improve consumption of unsaturated fat food sources such as sea fish, eggs, poultry, and soy protein; 3). To improve consumption of fruits and vegetables and 4). To limit consumption of fatty food or food processed with added fat such as palm oil or coconut milk. The participants recorded their attempts on a weekly monitoring form and discussed their progress with the facilitators at the end of the week. The delivery platform of the intervention was described thoroughly elsewhere [12].

### The Comparison Group

As a comparison group, the non-FBR group received an appropriate explanation of their dyslipidemia status at the beginning of the intervention. They also received once standard nutrition education by field nutritionists from primary health care either in a group or individually related to balance nutrition and dietary advice for dyslipidemia management, but without FBR provision. They were also informed and challenged to have a second lipid profile measurement after 12 weeks from baseline.

### Data collection and analysis

Data were collected before the intervention (baseline) and after the 12-week intervention (end line). Subjects’ sociodemographic data were collected only at the baseline, which included data related to age, household size, household income, education, occupation, and physical activity. These data were collected through a structured interview using a questionnaire.

Data on nutritional status were generated from anthropometric measurements of body weight, height, and waist circumference. Body weight was measured to the nearest 0.1 kg using a digital SECA flat scale for mobile use. Height was measured to the nearest 0.1 cm using a microtoise stature meter, whereas waist circumference was measured to the nearest 0.1 cm using the SECA ergonomic circumference measuring tape. All measurements were performed by trained enumerators following a standardized procedure. BMI was calculated based on body weight (kg) divided by height squared (m^2^). The prevalence of overweight and obesity was measured based on those with BMI ≥ 25.0 kg/m^2^, whereas abdominal obesity was determined based on a waist circumference of ≥80.0 cm. Changes in body weight (kg), waist circumference (cm), and BMI (kg/m^2^) from the baseline to the end of the 12-week intervention were calculated from measurements at the end line minus baseline measurements.

Lipid profiles covered data on TC, LDL, HDL, and TG. After overnight fasting, as much as 5 mL venous blood sampling was drawn. To ensure patient safety during dyslipidemia screening, blood sampling was conducted by professional phlebotomists and under the supervision of the health center medical team. Blood was taken after the subjects signed informed consent.

Total plasma cholesterol, HDL cholesterol, and TG were measured via the enzymatic colorimetric method with standardized procedures using the Selectra-E Analyzer, whereas LDL cholesterol was calculated using the Friedewald equation [14]. The classification guidelines were based on the National Cholesterol Education Program Adult Treatment Pane II [15], in which subjects with dyslipidemia were identified based on TC level ≥ 200 mg/dL, LDL cholesterol level ≥ 100 mg/dL, HDL cholesterol < 60 mg/dL, or TG ≥ 150 mg/dL. The Castelli index (TC/HDL cholesterol) was also calculated to measure atherogenic risk [14].

Data were analyzed using IBM SPSS software for Windows version 20. We conducted a univariate analysis to determine the distribution of values of each variable studied. Continuous variables were tested for data normality based on the Kolmogorov–Smirnov test. We recoded variables during the analysis process. Within-group differences before and after the intervention were analyzed using a paired *t*-test (normally distributed data) or Wilcoxon test (non-normally distributed data). We used the McNemar test to compare categorical data (prevalence). We used an independent *t*-test if the data were normally distributed and the Mann–Whitney *U* test for abnormally distributed data to measure between-group differences at the baseline.

To analyze the effect of the intervention on the outcome variables, we used a linear mixed model analysis with participants nested within-cluster. The goal of the analysis was to discover whether the FBR promotion is an important predictor for the end line nutritional status and lipid profile parameters after being adjusted with baseline values. We assumed that sample variation at baseline in terms of cluster and some confounding factors such as age and hormonal contraceptives might play an important role in the outcome variables. To do so, we included time (baseline, end line) as repeated measurement, intervention group (FBR, non-FBR), and potential confounding variables (age and hormonal contraception use) as the fixed factors. We also put the cluster and subject variation as a random effect in the model analysis.

### Ethical Consideration

Ethical approval was issued by the Ethics Committee of the Faculty of Medicine, Universitas Indonesia, on December 3, 2018 (reg no. 1269/UN2.F1/ETIK/2018). Recommendation for research was also obtained from the Provincial Government Board of West Sumatra (recommendation no. B.070/48-PERIZ/DPM&PTSP/I/2019) and the Padang City Review Board (recommendation no. 200.01.130/Kesbangpol/2019). All participants signed informed consent. The protocol of the study was registered and can be accessed online at ClinicalTrials.gov Protocol Registration and Result System ID: NCT04085874.

## Results

### Trials profiles

**Fig. 1:**
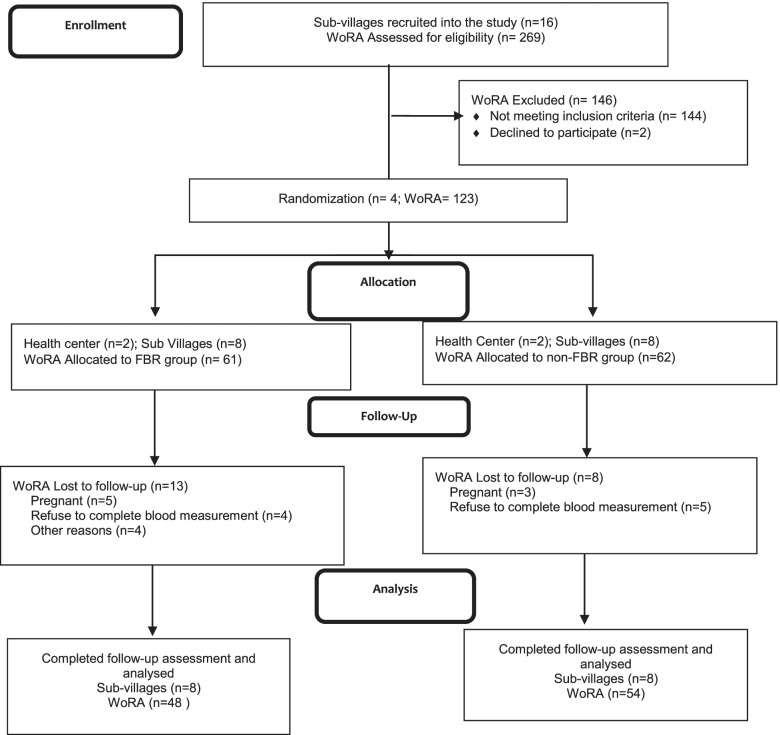
Adapted CONSORT diagram of the trial. FBR: Food-based Recommendation

Figure 1 shows the trial profile of the study. The screening process for subject recruitment was conducted between December 2018 and January 2019. A total of 123 subjects were eligible for the intervention; subjects were assigned to either the FBR group (n = 61) or the non-FBR group (n = 62). The intervention was administered over 12 weeks from the first week of February to the end of April 2020. Twenty-one participants (17%) dropped out of the study for several reasons, such as getting pregnant, not having enough time to stay in regular contact with promoters, feeling unable to follow the advice given in the intervention, and not having support from their family or husband. Therefore, a total of 102 subjects completed the intervention, comprising 48 and 54 WoRA in the FBR and non-FBR groups, respectively.

### Baseline characteristics of the participants

Table 1 summarizes the selected sociodemographic characteristics of the subjects at the baseline. The characteristics of the FBR and non-FBR groups were comparable, except for age, for which the median (min-max) age was higher in the FBR group than in the non-FBR group (39.5 [22–44] years and 35.5 [21–44] years, respectively). Most of the participants (56% and 70% in the FBR and non-FBR groups, respectively) had 12 years of schooling. Participants in the two groups predominantly worked as housewives. The median per capita monthly incomes of the subjects in the FBR and non-FBR group were IDR 525.000 and 500.000, respectively, which is equivalent to USD 1.2/cap/day. Most subjects engaged in moderate physical activity with a median parity of 2 (range, 0–6).

**Table 1:** Selected demographic and socioeconomic characteristics of the study subjects at the baseline

Parameter	Intervention Group		p-value*
	FBR	Non-FBR	
**Cluster level**			
Number of sub-villages	8	8	
Median (range) number of WoRA/sub villages	6 (3-9)	6(4-8)	
**Individual Level**			
Total participants completed the trial	48	54	
Age, years median (min–max)	39.5 (22–44)	35.5 (21–44)	**0.049**
Age category, n (%)			
20–34 years	14 (29.2)	25 (46.3)	
35–44 years	34 (70.8)	29 (53.7)	
Education, n (%)	12 (6–15)	12 (0–15)	0.178
No schooling	0 (0.0)	1 (1.9)	
Elementary	3 (6.2)	6 (11.1)	
Junior high school	10 (20.5)	8 (14.8)	
Senior high school	27 (56.2)	38 (70.4)	
Tertiary	8 (16.7)	1 (1.9)	
Occupation, n(%)			
Housewife	41 (85.4)	49 (90.7)	0.474
Government employee	1 (2.1)	0 (0.0)	
Small trader	3 (6.2)	1 (1.9)	
Others	3 (6.2)	4 (7.4)	
Marital status, n(%)			
Single	2 (4.2)	2 (3.7)	0.313
Married	44 (91.7)	52 (96.3)	
Widow	2 (4.2)	0 (0.)	
HH number, median (min–max)	5 (2–8)	4 (3–8)	0.636
Parity, median (min–max)	2 (0–6)	2 (0–6)	0.428
Per capita income, IDR median (min–max)	525.000 (200.000–3.000.000)	500.000 (180.000–1.466.666)	0.183
Physical activity, MET min/week	1242 (329–4617)	1257 (329–4518)	0.407
Physical activity category, n (%)			
Light	2 (4.2)	2 (3.7)	
Moderate	42 (87.5)	51 (94.4)	
High	4 (8.3)	1 (1.9)	
Having family history for NCDs, n (%)			
Heart diseases	5 (10.4)	4 (7.4)	0.731
Diabetes mellitus	12 (25.0)	15 (27.8)	0.751
Renal diseases	0 (0.0)	2 (3.7)	0.497
Hypertension	23 (47.9)	28 (51.9)	0.692
Stroke	4 (8.3)	5 (9.3)	1.000
Contraceptive user, n (%)	21 (43.8)	20 (37.0)	0.490
Hormonal contraceptives, n (%)	19 (39.6)	13 (24.1)	0.092

FBR, food-based recommendation; HH, household; MET, metabolic equivalent; NCD, noncommunicable diseases.

^*^Significant difference between the two groups: the Mann–Whitney analysis (for non-normally distributed continuous data) or chi-square analysis (for categorical data), significant at *p* < 0.05.

Table 2 shows the two groups were also comparable at the baseline in terms of body weight, height, BMI, and waist circumference (p > 0.05). Based on BMI, most participants were overweight or obese (81.3% and 75.3%, with mean ± SD of 28.7 ± 4.3 and 28.1 ± 4.6 kg/m2 in the FBR and non-FBR groups, respectively). Median Waist circumferences of the participants (min; max) in the FBR and the non-FBR group were 90.5(75;113) and 89.3(74;108) cm, respectively. Thus, most participants had a problem with abdominal obesity.

**Table 2:** The outcome variables characteristics of the study subjects at baseline

Parameters†	Units	Intervention Group		*p-*value between-group*
		FBR (n=48)	Non-FBR (n=54)	
Nutritional Status				
Body weight	kg	64.05(47.9;93.2)	64.7±12.2	0.283
Body Mass Index	kg/m^2^	28.7±4.3	28.1±4.6	0.693
Overweight/Obese	n (%)	39(81.3)	40(75.3)	0.389
Waist circumference	cm	90.5(75;113)	89.3(74;108)	0.634
Abdominal obesity	n (%)	41(85.4)	41(75.9)	0.230
Lipid Profiles				
Total cholesterol, (med, min-max) mg/dL	mg/dL	216 (162;277)	203(128;325)	**0.022**
LDL-cholesterol, (med, min-max) mg/dL	mg/dL	141(102;204)	132(73;257)	**0.010**
HDL-cholesterol, (med, min-max) mg/dL	mg/dL	42(34;58)	44(33;67)	0.153
Triglyceride, (median, min-max) mg/dL	mg/dL	123(65;391)	110(42;345)	0.121
Catelli’s Index I (TC/HDL) (med, min-max)	-	4.9(3.8;7.1)	4.5(2.7;7.7)	**0.020**
Castelli’s index I > 4.5	n(%)	33(68;7)	28(51.9)	0.083

FBR = Food Base Recommendations. LDL = Low density Lipoprotein; HDL = High Density Lipoprotein

†Continuous data are presented either in mean ± SD (normally distributed) or median (min; max) (not normally distributed). Categorical data are presented in n (%). ^*^Significant difference between the two groups, Man Whitney analysis (for not normally distributed continuous data) or Chi-square analysis (for categorical data), significant at *p*<0.05

However, the participants were not equally distributed into the FBR or non-FBR groups based on their lipid profile at baseline. Mean TC and LDL concentrations of the participants in the FBR group were significantly higher than those in the control group (p < 0.05). Meanwhile, HDL and TG levels, although not significantly different, were better in the non-FBR group. Most of the subjects in the two groups were classified as having dyslipidemia based on the abnormal levels of TC, LDL, or HDL. Only a few participants had a TG level above the cutoff point of 150 mg/dL. The Castelli index I (TC to HDL ratio), which implies atherogenic risk among the subjects in the intervention group, was significantly higher in the FBR than in the non-FBR group (p = 0.010). Approximately 68% of participants in the FBR group had a Castelli index greater than the cutoff of 4.5 (which implies high atherogenic risk), compared with approximately 52% in the non-FBR group.

### Changes in nutritional status

Table 3 shows the changes in nutritional status from baseline to the end of the 12-week intervention. Significant within-group improvements were observed in body weight, BMI, and waist circumference in the two groups (p < 0.001). Mean (95% CI) changes in body weight, BMI and waist circumference in the FBR group were -1.9 (-2.5; -1.4) kg, -0.80 (-1.03; -0.57) kg/m2, and -3.7(-4.7; -2.7) cm respectively, in comparison with -0.89(-1.3; -0.5) kg, −0.39(-0.57; -0.24) kg/m2, -1.9(-3.0; -0.97) cm in the non-FBR group. As the result, the prevalence of obesity (using the BMI and WC cut-off point) reduced 10.4 and 8 % respectively in the FBR group, as compared with only 1.8 and 0 % in the non-FBR group.

**Table 3:** Change of nutritional status from baseline at end-line (after 12-weeks intervention) by intervention group

Nutritional status Parameter/Indices	FBR Group (Intervention, n=48)			Non FBR (Comparison, n=54)			Mean difference† (FBR - Non-FBR) ((lower; upper bound of 95% CI), *p*-value*
	Baseline^¶^ (SE)	End of trial^¶^ (SE)	Change from baseline^¶^ (Lower; upper bound of 95% CI), *p*-value*	Baseline^¶^ (SE)	End of trial^¶^ (SE)	Change from baseline^¶^ (Lower; upper bound of 95% CI), *p*-value*	
Body weight, kg	67.6(1.69)	65.69(1.63)	-1.9(-2.5; -1.4), <0.001	64.9(1.82)	64.0(1.78)	-0.89(-1.3; -0.5), <0.001	-1.1(-1.86 ;-0.39;), **0.003**
Body Mass Index, kg/m^2^	28.8(0.64)	27.9(0.62)	-0.8(-1.03; -0.57), <0.001	28.2(0.62)	27.8(0.62)	-0.39(-0.57; -0.24), <0.001	-0.43(-0.76; -0.11), **0.008**
Waist circumference, cm	90.9(1.32)	87.2(1.34)	-3.7(-4.7; -2.7), <0.001	89.8(1.33)	87.9(1.33)	-1.9(-3.0; -0.97), <0.001	-2.1(-3.7; -0.46), **0.013**
Overweight and obesity, n (%)	39(81.3)	34 (70.9)	-5(-10.4), 0.046	40(75.3)	39(72.3)	-1(-1.8). 0.3117	n.a
Abdominal obesity, n (%)	41(85.4)	37 (77.1)	-4(-8.3), 0.157	41(75.9)	41(75.9)	0(0.0), 1.00	n.a

FBR = Food Base Recommendations; SE = standard error; n.a = not available

^¶^Adjusted for age, contraception use, and stratification variables at randomization (subject nested within the cluster) using Mixed Model Analysis

†Adjusted for age, contraception use, and stratification variables at randomization (subject nested within the cluster) using Mixed Model Analysis

*Significant difference at p<0.05

By using the linear Mixed Model analysis we observed significant changes in the body, BMI, and waist circumference weight (p < 0.001) as the effect of the intervention. The mean effect of the intervention on body weight, BMI and waist circumference for the FBR group versus the non-FBR group were -1.1 (-1.8; -0.39) kg, -0.43(-0.76; -0.11) kg/m2 and -2.1(-3.7;-0.46) mm respectively (p <0.05). The model analysis also informs that changes in the nutritional status were not associated with age and hormonal contraception use *or* occurred by chance due to variation of subject and the cluster (p>0.05).

### Changes in lipid profiles

Table 4 presents the effect of the intervention on the subjects’ lipid profiles. At the end of the trial, we observed the same pattern of increase in TC, LDL, and HDL but a decrease in TG and Castelli index in the two groups. Mean (95% confidence interval) changes in TC, LDL, HDL, and TG were 12.5(7.0;14.8) mg/dL, 9.8(4.7;14.8) mg/dL, 5.0(3.2;6.9) mg/dL, and −16.9 (-35.4;-1.9) mg/dL, respectively in FBR group, in comparison with 15.5 (10.5;20.6) mg/dL, 14.6(4.7; 14.8) mg/dL, 4.0(1.9; 6.1) mg/dL, and −15.7(−26.3; -5.1) mg/dL, respectively, in the non-FBR group. Changes in the lipid parameters significantly affect the total cholesterol to HDL ratio (Castelli index I) in the FBR group (p<0.005), but not in the non-FBR group (p>0.05). The mean (95% confidence interval changes in the Castelli index in the FBR group was −0.22(−0.4; -0.05), in comparison with −0.1 (-0.35; 0.14) in the non-FBR group. The prevalence of participants with a Castelli index I > 4.5 decreased 18.7% in the FBR group compared with 14.8% in the non-FBR group. As much as 70.8% and 66.7% of respondents in the FBR and non-FBR groups, respectively, experienced a decrease in the atherogenic index.

**Table 4:** Change of lipid profiles from baseline at end-line (after 12-weeks intervention) by intervention group

Lipid profile parameters	FBR Group (Intervention, n=48)			Non FBR (Comparison, n=54)			Mean difference† (FBR - Non-FBR) ((lower; upper bound of 95% CI), p value*
	Baseline^¶^ (SE)	End of trial^¶^ (SE)	Change from baseline^¶^ (Lower; upper bound of 95% CI), *p*-value*	Baseline^¶^ (SE)	End of trial^¶^ (SE)	Change from baseline^¶^ (Lower; upper bound of 95% CI), *p*-value*	
Total cholesterol, mg/dL	221 (4.1)	230 (3.9)	12.5(7.0; 14.8), **0.006**	207(4.9)	222(4.6)	15.5(10.5;20.6), **0.045**	-2.9(-9.7; 3.86), 0.391
LDL-cholesterol, mg/dL	149 (3.9)	155 (3.5)	9.8(4.7;14.8), **0.033**	135(4.3)	150(4.2)	14.6(4.7;14.8), 0.064	-4.8(-11.1;1.4), 0.129
HDL-cholesterol, mg/dL	44.2 (1.6)	49.3(2.1)	5.0(3.2;6.9), **<0.001**	46(1.2)	50(1.7)	4(1.9; 6.1), **<0.001**	0.4(-2.7;3.7), 0.767
Triglyceride, mean (SE), mg/dL)	139 (12.8)	122(11.5)	-16.9(-35.4; 1.9), 0.077	126(8.6)	111(7.8)	-15.7(-26.3; -5.1);**0.005**	-2.4(--26.8;21.9), 0.843
Catelli’s Index I(TC/HDL)	5.0 (0.118)	4.8(0.155)	-0.22(-0.4; -0.05), **0.011**	4.57(0.13)	4.47(0.18)	-0.1(-0.35;0.14); 0.397	-0.19(-0.544; 0.167), 0.294
Catelli’s Index II(LDL/HDL)	3.4 (0.10)	3.3(0.13)	-0.11(-0.28;0.04), 0.147	2.9(0.11)	2.9(0.16)	0.001(-0.24; 0.24), 0.991	-0.10(-0.56; 0.11), 0.178
Castelli’s index I > 4.5, n (%)	33 (68.7)	24(50.0)	-9(-18.7)	28(51.9)	20(37.0)	-8(-14.8)	n.a
Decrease in Castelli’s Index, n (%)	n.a	34(70.8)	n.a	n.a	36(66.7)	n.a	n.a

FBR = Food Base Recommendations; LDL = Low density Lipoprotein; HDL = High Density Lipoprotein; n.a = not available

^¶^Adjusted for stratification variables at randomization (cluster) using Mixed Model Analysis

†Adjusted for age, hormonal contraception use, and stratification variables at randomization (subjects nested within the cluster) using Mixed Model Analysis

*Significant difference at p<0.05

The mean (95% confidence interval) effect of the intervention on TC, LDL, HDL, and TG for the FBR group versus the non-FBR group were -2.9(-9.7;3.96) mg/dl, -4.8(-11.1;1.4) mg/dl; 0.4(-2.7;3.7) mg/dl, and -2.4(-26.8;21.9) mg/dl respectively. The mean between-group difference (95% confidence interval) of TC to HDL and LDL ratio were -0.19(-0.54;0.167) and -0.10(-0.56;0.11) respectively. Although greater improvements of lipid profile were observed in the FBR group, there was no significant impact of the intervention (p>0.05).

The mixed model analysis reveals that lipid profiles were not significantly affected by group (intervention) and subject cluster (p>0.05), but it was predominantly influenced by age and the use of hormonal contraceptives (p<0.05). From the covariant parameters estimates, it was noticed the older the age the higher the TC, LDL, and TG, while hormonal contraceptive use constantly caused an increase in TC, LDL, and TG levels.

## Discussion

In response to a nutritional intervention promoting the optimized FBRs, this study found significant improvements in body weight, BMI, and waist circumference. Improvements in nutritional status occurred in both intervention groups, but a greater increase occurred in the FBR group (*p* = 0.001). Considering that most respondents in the two groups were categorized as overweight or obese at the baseline, they were motivated during the intervention to monitor their weight regularly. However, those in the FBR group were strongly advised to reduce consumption of energy-dense foods, especially flour-based, high-sugar, and fatty foods which are important to control energy intake in weight management.

When compared with a diet program for weight loss, the FBR promotion had a relatively lower effect on changes in body weight. Using a diet program, normal weight loss ranges from 0.5 to 1.0 kg per week [16] or approximately 6–12 kg for 12 weeks. This may be related to the improvement in dietary practices and small changes in energy intake. The FBRs were not designed to be a weight loss program that was restrictive regarding the quantity of energy, fat, protein, and carbohydrate. Rather, they intended to fulfill energy and nutrient requirements based on ideal body weight as well as to improve dietary composition. Therefore, the energy requirement used in the LP analysis was based on ideal body weight (54 kg), which is lower than the subjects’ median body weight (67 kg). However, ultimately, the balanced diet indirectly had a positive effect on weight maintenance, although it took relatively longer. Even small amounts of weight loss bring health benefits, including lowered cardiovascular risk, prevention, delayed progression, or improved health conditions [16].

Based on the indicators of dyslipidemia (TC, LDL, HDL, and TG levels), this study found that a 12-week promotion of FBRs did not have a significant effect on the improvement of lipid profiles. One interesting fact that we found was that two of the four parameters (TC and LDL levels) worsened, whereas we improved the other two parameters (HDL and TG) at the end of the intervention. The changes occurred in the same pattern in both intervention groups. However, when we examine the Castelli index (TC to HDL ratio), there was a greater reduction in atherogenic risk in the FBR group than in the non-FBR group, although this difference was not statistically significant.

The improvement in HDL and triglyceride levels may be the result of increased consumption of vegetables, fruit, and fish containing omega 3 and omega 6 fatty acids. As we reported in the previous article, these foodstuffs were food ingredients that were promoted during the promotion of FBR [12]. This is in line with a study with the provision of a Mediterranean diet (low energy from saturated and trans fatty acids; high omega-3 fatty acids intake of plant and marine origin; increase intake of fruits, vegetables, and soluble fiber) has an HDL level 2-3 mg/dL higher and TG levels 13 mg/dL lower than the control group [17].

When we calculated the effect size of the intervention on lipid parameters based on the mean or median change [18] of respective parameters between-group differences (0.2 = small, 0.5 = medium, 0.8=high), this present study found a relatively smaller effect size (0.01–0.24) as compared with several previous interventions. An intervention study using the Dietary Approach to Stop Hypertension (DASH) diet for 8 weeks provided an effect size of 0.26–0.4 on the lipid profile, and a diet high in fruits and vegetables had a lower effect size (0.09–0.12) than the DASH diet had [19]. The greater effect size on HDL and TG was shown by the intervention on obese subjects using a monthly counseling session on consuming a reduced-calorie diet and engaging in physical activity for 2 years (the effect size was 0.67 and 0.31 for HDL and TG, respectively) [20]. Another study that provided weekly nutrition education, combined with a three-times-per-week exercise program for 12 weeks also showed a small to medium effect size (0.45–0.51) on TC, LDL, HDL, and TG [21]. A study that provided a low-fat, low-cholesterol, high-fiber diet showed a greater effect size (0.59–1.2) on TC, LDL, and HDL.

However, the intervention design of the previous studies differed from that of the present study, wherein the interventions provided were in the form of more structured meal provision, such as the proportion of energy from macronutrients, the composition of fatty acids, and the total amount of energy intake, followed later by the provision of a regular exercise program. Meanwhile, the intervention in the present study was in the form of only FBR promotion on ideal dietary practices, but there was no provision of any food or exercise program. Decisions regarding food choices and consumption, as well as whether to engage in physical exercise during the intervention, were made entirely by the respondents. Thus, an education program with a longer period of intervention, combined with the provision of a low-fat and high-fiber diet, and the addition of the exercise program could have a better impact than a single FBR promotion on the subject’s lipid profile.

The same pattern of the increase of TC and LDL in the two groups implies that other existing factors in the two intervention groups might have produced a greater impact on lipid profile rather than the intervention. It has been documented that approximately 60%–75% of blood cholesterol is determined by body metabolism or endogenous pathway, whereas this figure is only approximately 25%–40% for dietary cholesterol [22]. Furthermore, previous studies have confirmed that internal factors such as age and hormonal contraception significantly influence the lipid profiles among women [23–26].

Participants in this study were mostly older than 35 years, and those in the FBR group were significantly older (by 4 years) than those in the non-FBR group. Among the two groups in this study, approximately 30% used hormonal contraceptives, which was believed to contribute significantly to the increase in TC and LDL levels in the two groups at the end of the intervention. When we analyzed differences in lipid profiles between the hormonal contraception user and the non–hormonal contraception users, we found a significant between-group difference (*p* < 0.001) in changes in TC and LDL levels. The mean ± SD increase in TC and LDL levels in the hormonal contraception user group was 26 ± 15.5 and 25.8 ± 14.9 mg/dL, respectively, in comparison with −1.49 ± 16 and −3.4 ± 14.8 mg/dL in the non–hormonal contraception user group. The effect of age and the use of hormonal contraceptives on end-line lipid profiles in this study were significantly consistent in the mixed model analysis (p<0.005).

Apart from these risk factors, initial baseline lipid profiles between the two groups might have influenced the effect of the intervention on the outcome. As we can see from the baseline characteristics, the participants in the FBR group had lipid profiles worse than those in the non-FBR group. When stratified based on the median Castelli index at the baseline, we found that 14 of 48 subjects (29%) in the FBR group experienced an increase in atherogenic risk at the end of the intervention. Among these subjects, 10 (71%) are those with a baseline Castelli index greater than the median. Meanwhile, in the non-FBR group, 18 of 54 subjects (33%) also experienced an increase in the Castelli index; of them, 45% had a Castelli index greater than the median at the baseline.

Another factor that might be related to the increase in TC and LDL cholesterol in both groups is the type of cooking oil used in food processing, which has an impact on the increased intake of saturated fatty acids and trans fatty acids. Palm oil was the most available and affordable cooking oil and was used mostly in frying. The use of palm oil for frying is believed to have a worse impact than the use of coconut or coconut oil in food processing. This is related to the composition of fatty acids in palm oil, approximately 50% of which is unsaturated fatty acids [27]. The high temperature during frying causes unsaturated fatty acids to transform into trans fatty acids, which play a role in increasing blood cholesterol [28]. Unfortunately, our qualitative data showed that our subjects mostly used unpackaged palm oil, locally called “bulk oil,” which has no quality control during the process of production and distribution. It is also likely to be mixed with reused cooking oil, which has deleterious health effects [29]. Furthermore, it is also common in the community to reheat fried foods, which results in increased consumption of saturated fatty acids. This is consistent with the findings of previous studies, in which an increased intake of saturated fat and trans-fatty acids worsened the LDL cholesterol level [29, 30].

Other factors that might influence changes in lipid profile such as genetics and stress level were unfortunately not measured in this study. Previous studies have found that the presence of modalities in the distribution of LDL particle size indicates possible effects of the main genes underlying them, although the mode of transmission and specific genes involved have not been precisely determined [32]. Several studies have shown the effect of stress on lipid metabolism. Stress associated with a major disaster, such as an earthquake or loss of a job and income, is associated with increased bloodstream levels of TC, LDL, and TG [33]. The perception of increased stress during a period of high workload is associated with elevated cholesterol in the bloodstream and ingestion of foods that increase cholesterol [34]. Dyslipidemia induced by stress is part of the body’s response to cope with stressors. When the stressor is maintained over a long period, stress-induced dyslipidemia persists and may have deleterious effects [35].

Although we did not find a significant effect of the intervention on lipid profiles, HDL levels improved significantly in both groups, which affected Castelli index reduction in both groups. The decline in the index provides information regarding reducing the atherogenic risk in the subjects in both groups. The improvement in the index in the FBR group was greater than that in the non-FBR group. This was clinically important, considering that the mean (95% CI) of Castelli Index I in the FBR group reduced by 0.22 (0.05 – 0.4) points. This demonstrates that the use of the Castelli index could enable a more thorough evaluation of the impact of an intervention on dyslipidemia, whereas the use of single indicators such as TC, LDL, HDL, or TG alone could lead to a less comprehensive interpretation of the patient situation.

The results of this study are in line with those of several previous publications, which proposed the use of the Castelli index as a better diagnostic alternative in predicting the risk of developing cardiovascular events because it incorporates information on both LDL and TG in the numerator and HDL as the denominator [35, 36]. An increase in TC and LDL, as found in this study, does not necessarily increase the atherogenic risk when accompanied by an increase in HDL and a decrease in TG. Scientific evidence shows that an increase in HDL levels is profoundly antiatherogenic and is an inverse predictor of cardiovascular disease. HDL particles have several functions with the potential to protect against arterial disease, which is related to their ability to promote cholesterol efflux from macrophages in the artery wall and reduce oxidation [38].

To the best of our knowledge, the present study is the first to evaluate the efficacy of an optimized FBR promotion in improving the nutritional status and lipid profile among women of reproductive age with dyslipidemia. We found that the intervention significantly improved nutritional status and also had a minor effect on lipid profile and reduction of atherogenic risk. One limitation of this study was related to the significant difference in some of the baseline parameters, especially lipid profile indictors, between the FBR and non-FBR groups. We used a cluster-randomized community trial, as this was the best study design to be applied for the FBR promotion. However, the randomization result was not as expected, as there was a lack of between-group comparability in some parameters at the baseline.

## Conclusions

The promotion of community-based FBRs produced a strong improvement in nutritional status. Marginal but significant decreases in body weight, BMI, and waist circumference occurred as a result of the intervention. The small effect size was observed in the improvement of the Castelli index, although these changes were not statistically significant. We suggest that future studies should explore the effect of an FBR promotion for a longer intervention period or combine the FBR promotion with the provision of a physical activity program. Operational research is also required to explore some alternatives for implementing the FBR promotion within the content of a health nutrition system.

## Data Availability

The datasets generated and/or analyzed during the current study are not publicly available due to the data set of this study being the property of The University of Indonesia but are available from the corresponding author on reasonable request.

## Abbreviations

BMI: Body Mass Index
FBR: Food-Based recommendation
DASH: Dietary Approach to Stop Hypertension
HDL: High Density Lipoprotein
LDL: Low Density Lipoprotein
LP: Linier Programming
PUFA: Poly Unsaturated Fatty Acids
SAFA: Saturated Fatty Acids
TC: Total Cholesterol
TG: Triglycerides
